# Composition and natural history of the snakes from the Parque Estadual da Serra do Papagaio, southern Minas Gerais, Serra da Mantiqueira, Brazil

**DOI:** 10.3897/zookeys.797.24549

**Published:** 2018-11-19

**Authors:** Frederico de Alcântara Menezes, Arthur Diesel Abegg, Bruno Rocha da Silva, Francisco Luís Franco, Renato Neves Feio

**Affiliations:** 1 Universidade Federal de Viçosa, Departamento de Biologia Animal, Avenida P.H. Rolfs, S/N, Campus Universitário, CEP 36571-000, Viçosa, MG, Brazil Universidade Federal de Viçosa São Paulo Brazil; 2 Instituto Butantan, Laboratório Especial de Coleções Zoológicas, Avenida Vital Brasil, 1.500, Butantã, CEP 05503-900, São Paulo, SP, Brazil Instituto Butantan, Laboratório Especial de Coleções Zoológicas São Paulo Brazil

**Keywords:** Araucaria Forests, Atlantic Rainforest, cluster analysis, Serpentes, southeastern Brazil

## Abstract

The Serra da Mantiqueira is one of the least inventoried physiographic areas of southeastern Brazil. There is great potential for detection of endemic species for which little or nothing is known about basic aspects of natural history. The Parque Estadual da Serra do Papagaio (PESP) within the Serra da Mantiqueira is an area of extreme biological importance because it houses mixed formations of grasslands, ombrophilous forests, and enclaves of Araucaria forests (mixed ombrophilous forest). Currently, the mixed ombrophilous forest covers less than 5% of its original range and areas occupied by this forest type, and associated ecosystems constitute refuges, housing several endemic, high altitude species. Between September 2015 and April 2016, field samplings were performed in the PESP using four distinct methods. The objective was to determine the composition and natural history of snakes from an isolated, high altitude area of the Serra da Mantiqueira. In PESP and surrounding areas, 80 individuals representing 24 species, 19 genera, and three families were recorded. Data are presented on abundance, habitat, daily activity, diet, reproduction, and defense. Comparison of the PESP snake assemblage with 30 other Atlantic Forest areas in southeastern Brazil indicate the Serra da Mantiqueira presents particular characteristics regarding snake composition.

## Introduction

The structure of a snake community can be influenced by historical factors, such as biogeography (Cadle and Greene 1993, Martins and Oliveira 1998, Marques 1998, Sawaya et al. 2008), and ecological factors, such as competition (Inger and Colwell 1977, Henderson et al. 1979, Vitt and Vangilder 1983, Pianka 1989, Krebs 2001), predation (Connel 1975, Krebs 2001), and the influence of species natural history on the assemblage evolution (Schoener 1968, 1983, Wiens 1977). However, the basic natural history and biology for many snake species remains unknown. This is compounded by loss of such information with the reduction of habitat (Marques 1998). Description of snake communities with the intention of understanding interactions between species, patterns of diversity (e.g., species richness, dominance and relative abundance), and the processes that influence community structure is becoming increasingly important.

Snake fauna of tropical areas is typically characterized by high species richness, low abundance, and complex ecological interactions (Duellman 1978, Henderson et al. 1979). These features pose challenges to quantitative and comprehensive study of these populations. There have been several efforts to describe the ecology and natural history of snakes in Brazil (e.g., Amazon Forest: Cunha and Nascimento 1978, Martins and Oliveira 1998, Pantanal: Strüsmann and Sazima 1993, Cerrado: Nogueira 2001, Sawaya et al. 2008, Caatinga: Mesquita et al. 2013, Guedes et al. 2014; Atlantic Forest: Marques 1998, Cicchi et al. 2007, Centeno 2008, Cicchi et al. 2009, Hartmann et al. 2009a, b, Trevine et al. 2014). Although these studies have advanced our understanding of snake fauna in various ecosystems, studies focusing on snake communities in high altitude areas and the Araucaria forests remain scarce. Studies with this focus are mainly concentrated in the Serra do Mar of São Paulo State (e. g., Parque Estadual da Serra do Mar: Hartmann et al. 2009b; Parque Natural Municipal Nascentes de Paranapiacaba: Trevine et al. 2014; Parque Nacional da Serra da Bocaina: Ortiz et al. (2017) and southern Brazil (e.g., Centro de Pesquisas e Conservação da Natureza Pró-Mata: Di-Bernardo 1998; Parque Nacional de Aparados da Serra: Deiques 2009). However, there is a lack of knowledge about the Serra da Mantiqueira snake fauna.

The Serra da Mantiqueira is one of the least well-understood physiographic areas of southeastern Brazil. There is considerable potential for the record of remarkable and endemic species, for which basic natural history has yet to be described. To date only a single study reports on a snake assemblage in this region (Cardoso 2011). This work presented the ecological aspects of a snake community in Munhoz (southern Minas Gerais) and provided the first insight into the composition and natural history of snakes in this region. Here we report on the snake composition of the Parque Estadual da Serra do Papagaio (PESP), Minas Gerais, Southeastern Brazil, a high altitude area of the Serra da Mantiqueira. We describe the species observed, natural history data, altitudinal distribution, as well as an identification key for the recognition of snake species in the area. Finally, we compare the snake fauna composition of PESP with those of 30 other Atlantic forest areas in southeastern Brazil, including the states of Minas Gerais (MG), Rio de Janeiro (RJ) and São Paulo (SP).

## Materials and methods

### Study area

This study was conducted in the Parque Estadual da Serra do Papagaio (PESP) (22°8'33"S, 44°43'43"W, ca. 22.900 ha) located at Serra da Mantiqueira, southern Minas Gerais State, southeastern Brazil. The PESP overlaps the municipalities of Aiuruoca, Alagoa, Baependi, Itamonte and Pouso Alto within MG, and has an altitudinal range from 1200–2359 m above sea level (Silva et al. 2008). Southward, the PESP contacts the Itatiaia National Park, forming an ecological corridor between the forests of southern Minas Gerais with those of the coastal mountain ridges (= Serra do Mar) in Rio de Janeiro and São Paulo States. The park presents little-disturbed vegetation, within five vegetation zones: 1 - Ombrophilous Dense High Montane Forest (nebular forest), covering altitudes above 1800 m a.s.l.; 2 - Ombrophilous Dense Montane Forest, covering altitudes below 1800 m a.s.l.; 3 - Mixed Ombrophilous Forest, concentrated in valleys along watercourses at 1600 m a.s.l.; 4 – High altitude grasslands, covering parts with altitudinal heights between 1300 and 1800 m a.s.l. and 5 - Rocky Fields, associated with rocky outcrops above 2000 m a.s.l. (Silva et al. 2008). According to Köppen`s classification, the predominant climate is mesothermal tropical of altitude, with a cold and dry winter, and high rainfall levels in the summer. The average annual precipitation exceeds 1500 mm, with 80% occurring from October to March. Winter temperatures range from 0 °C to 10 °C and frost and drought can occur during in this period. Summer is mild with temperatures ranging up to 30 °C (Silva et al. 2008).

### Sampling design

Snake sampling lasted eight months, from September 2015 to April 2016. We sampled between altitudes 1600 and 2359 m a.s.l. across all of the available vegetation types. Field trips of four to seven days at a time were made monthly for a total of 40 days of field observation. Four different sampling methods were used to capture snakes:

1) Pitfall traps with drift fences (Greenberg et al. 1994, Cechin and Martins 2000) were installed in three types of vegetation: Ombrophilous Dense Montane Forest, Mixed Ombrophilous Forest, and High Altitude Grasslands. Two sets of traps were used, each comprised of two 50-meter lines, separated from each other by 100 m. Each line consisted of five 60-L buckets, joined by an approximately 50 cm high drift fence. The buckets were drilled in the bottom to avoid accumulation of rainwater. Inside each bucket, we also put foliage together with a styrofoam plate to serve as a refuge for the fallen animals (Mesquita et al. 2013). The drift fence was buried 20 cm below the ground and held upright by wood stakes. These traps were opened for the four to seven days of each monthly field trip, except for May, June, July, and August. They were inspected daily, totaling 40 days of open traps (2400 bucket days).

2) Time-constrained search was also employed (Campbell and Christman 1982, Martins and Oliveira 1998). Trails were covered on foot, searching all possible shelters and microhabitats that might be used by snakes. In total, 360 hours of visual searching was performed in the study area vegetation types.

3) Accidental encounter (Sawaya et al. 2008) of live or dead specimens sampled opportunistically, with no methodology like pitfall traps or time-constrained search. Here we included individuals found in the PESP and the surrounding areas. Snakes found in the surroundings were included in the list only when recorded above 1600 m (lower altitude of our sampling site) and if they presented literature records at equal to or higher elevations. This methodology was used primarily on roads BR 354, LMG 881, and an unpaved road that links Itamonte, MG, to the PESP headquarters.

4) Records made by local people (Martins and Nogueira 2012). Snakes found by local people from the PESP area and surroundings were also incorporated in the sampling. To get more information about the specimens found, we delivered record sheets for registering information on snakes, such as time and site of the encounter (open area, forest edge, and forest interior), behavior and posture (moving, stationary, coiled or stretched).

For each specimen, we recorded: date and time of observation, habitat, microhabitat, mass (g), sex, diet, reproduction, activity, and defensive behavior. Snake size was categorized according to Marques et al. (2001). Diet was characterized in two different ways. Collected snakes had their stomach examined through a ventral incision along the posterior two-thirds of the body (Martins and Gordo 1993). However, most individuals were not collected and so they were submitted to regurgitation through soft palpation on the abdomen in an antiperistaltic movement (Shine 1995). These animals were subsequently released at the site of capture. Whenever diet items were found, they were identified to the lowest possible taxonomic level using identification keys, comparison with other specimens from zoological collections, and expert assistance. To describe the reproductive condition, we recorded the number of follicles in secondary vitellogenesis or eggs/embryos present in females (Almeida-Santos et al. 2014). The specimens collected during this study were deposited in two zoological collections: Coleção Herpetológica Alphonse Richard Hoge of Instituto Butantan (IBSP), São Paulo, SP, Brazil and in Museu de Zoologia João Moojen (MZUFV), Viçosa, MG, Brazil.

### Data analysis

**Figure 1.**
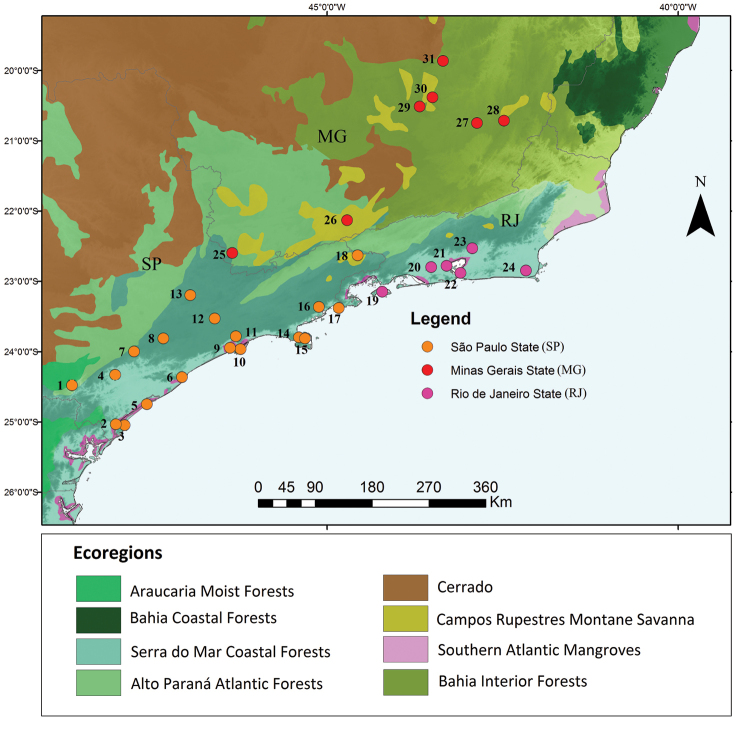
Areas used for the analysis of similarity between snake assemblages.The following snake assemblages were included in the analyzes: São Paulo State: **1** Parque Estadual Turístico do Alto Ribeira (Araújo et al. 2010) **2** Ilha da Cananéia (Cicchi et al. 2007) **3** Parque Estadual Ilha do Cardoso (Rocha et al. 2008) **4** Fazenda Etá (Fiorillo 2016) **5** Ilha Comprida (Cicchi et al. 2007) **6** Estação Ecológica Juréia-Itatins (Marques and Sazima 2004) **7** Parque Estadual Carlos Botelho (Forlani et al. 2010) **8** Municipalities of Tapiraí and Piedade (Condez et al. 2009) **9** São Sebastião (Centeno et al. 2008) **10** Ilhabela (Centeno et al. 2008) **11** Parque Municipal de Paranapiacaba (Trevine et al. 2014) **12** São Paulo (Barbo et al. 2011) **13** Parque Estadual da Serra do Japi (Sazima and Haddad 1992) **14** São Vicente Island (Cicchi et al. 2007) **15** Santo Amaro Island (Cicchi et al. 2007) **16** Parque Estadual da Serra do Mar (Núcleo Santa Virgínia) (Hartmann et al. 2009a) **17** Parque Estadual da Serra do Mar (Núcleo Picinguaba) (Hartmann et al. 2009b) **18** São José do Barreiro (Ortiz et al. 2017); Rio de Janeiro State **19** Ilha Grande (Rocha and Van-Sluys 2006) **20** Parque Natural Municipal da Serra do Mendanha (Pontes et al. 2009) **21** Duque de Caxias (Salles and Silva-Soares 2010) **22** Niterói (Citeli et al. 2016) **23** Estação Ecológica do Paraíso (Vrcibradic et al. 2011) **24** Núcleo Experimental de Iguaba Grande (Martins et al. 2012) Minas Gerais State **25** Munhoz (Cardoso 2011) **26** Parque Estadual da Serra do Papagaio (This study) **27** Parque Estadual da Serra do Brigadeiro (Moura et al. 2012) **28** Viçosa (Costa et al. 2010) **29** Ouro Branco (São-Pedro and Pires 2009) **30** Ouro Preto and surroundings (Silveira et al. 2010) **31** Estação Ambiental de Peti (Bertoluci et al. 2009).

We compared the snake assemblage from Parque Estadual da Serra do Papagaio with those of 30 other localities in the Atlantic forest, in southeastern Brazil (Fig. 1).

With this data set, we generated a binary presence/absence matrix of 120 species. To compare snake assemblages, we used this matrix to run a Cluster Analysis, using the Jaccard’s similarity index, and the Pair Group Average Method (UPGMA) as the grouping method. We also calculated the cophenetic correlation coefficient to indicate the similarity matrix degree of representation in the dendrogram. In this index, values greater than or equal to 0.8 allow considering the dendrogram as adequate to the similarity matrix (Rohlf 2000). The result of this analysis was visually compared, allowing the identification of groups clustered together by the similarities of species composition from different localities.

Finally, a Nonmetric Multidimensional Scaling (NMDS) was used for another view of the Jaccard index clusters. The stress value was used as a representative measure of the groupings, and values <0.20 were considered acceptable (Clarke and Warnick 1994). Multivariate analyzes were performed in R software (R Core Team 2014), using the *vegan* package (Oksanen et al. 2015).

### Taxonomic accounts

Several nomenclature changes have been proposed to the taxonomy of Neotropical snakes in recent years. To provide consistency during similarity analysis we present a brief list of species that have had names changes and identify the name used in this report. The name “Taeniophallusgr.occipitalis” was used for specimens traditionally referred to as *Taeniophallusoccipitalis* (Jan, 1863) because more than one species often fall under this designation (Santos-Jr. 2008). Specimens identified as *Mussuranamontana* (Franco, Marques & Puorto, 1997) by Hartmann et al. (2009b) belong to *Pseudoboaserrana* Morato, Moura-Leite, Prudente & Bérnils, 1995 (Francísco Luís Franco pers. obs.). Specimens identified as *Atractus* sp. by Hartmann et al. (2009b) were described as *Atractusfrancoi* Passos, Fernandes, Bérnils and Moura-Leite, 2010. Specimens referred to as *Dipsas* sp. by Hartmann et al. (2009a) were described as *Dipsassazimai* Fernandes, Marques & Argôlo, 2010. Specimens identified as *Dipsasindica* Laurenti, 1768 by Hartmann et al. (2009a) were considered *Dipsaspetersi* Hoge & Romano, 1975, according to Harvey and Embert (2008). The specimen referred to as *Helicops* sp. by Costa et al. (2010) was described as *Helicopsnentur* Costa, Santana, Leal, Koroiva and Garcia 2016. The specimen identified as Tropidophiscf.paucisquamis by Silveira et al. (2010) was described as *Tropidophispreciosus* Curcio, Nunes, Argôlo, Skuk & Rodrigues, 2012. The specimen identified as *Philodryasoligolepis* Gomes *in* Amaral, 1921 by Silveira et al. (2010) belongs to *Philodryaslaticeps* Werner, 1900 (Zaher et al. 2008). Specimens identified as *Thamnodynastes* sp. by Salles and Silva-Soares (2010) belong to *Thamnodynastesnattereri* (Mikan, 1820) (Franco and Ferreira 2002), as well as the specimens cited as Thamnodynastescf.nattereri by Marques and Sazima (2004), Centeno et al. (2008), Pontes et al. (2008), Bertoluci et al. (2009), Hartmann et al. (2009b), Silveira et al. (2010), Vrcibradic et al. (2011), Moura et al. (2012) and Citeli et al. (2016). Specimens considered as *Chironiusflavolineatus* (Jan, 1863) by Condez et al. (2009), São Pedro and Pires (2009), Forlani et al. (2010) and Silveira et al. (2010) were described as *Chironiusbrazili* Hamdan & Fernandes, 2015 (Hamdan and Fernandes 2015). The specimen considered *Micrurusibiboboca* (Merrem, 1820) by Martins et al. (2012) corresponds to a new species which is being described (Francisco Luís Franco, unpubl. data). The specimen regarded as *Dipsasincerta* (Jan, 1863) by Citeli et al. (2016) corresponds to *Dipsasalternans* (Fischer, 1885) (Passos et al. 2004). The specimen cited as *Epicrates* sp. by Moura et al. (2012) was considered *Epicratescenchria* (Linnaeus, 1758) according to the own comments on Moura et al. (2012). Specimens addressed as *Dipsasneivai* Amaral, 1926 by Centeno et al. (2008) correspond to *Dipsasvariegata* (Duméril, Bibron & Duméril, 1854) (Harvey and Embert 2008). The specimens cited as Chironiuscf.quadricarinatus and Liophiscf.almadensis by Bertoluci et al. (2009) were considered *Chironiusquadricarinatus* (Boie, 1827) and *Erythrolamprusalmadensis* (Wagler, 1824), respectively. Specimens identified as Tantillacf.melanocephala by Barbo et al. (2011) are here considered *Tantillamelanocephala* (Linnaeus 1758). The specimen “IBSP 30496” regarded as *Cleliarustica* (Cope, 1878) by Franco et al. (1997), corresponds to a new species that is being described (Francisco Luís Franco, unpubl. data). Taxonomy on family level follows Grazziotin et al. (2012).

## Results

### Species composition

We recorded 80 snakes during the eight month period of fieldwork through all sampling methods. In this group we separated 24 species of 19 genera, of which 67% are dipsadids, 19% viperids, and 14% colubrids (Table 1). The five most abundant species in the study area were dipsadids *Atractuszebrinus* and *Thamnodynastesstrigatus* (each 18.5% of the records), followed by *Philodryaspatagoniensis* (11.2%), *Gomsesophisbrasiliensis* (10%), and the viperid *Bothropsfonsecai* (8.7%). Among viperid records, the most abundant species was *B.fonsecai* (*n* = 7; 63%), followed by *B.jararaca* (*n* = 2; 18%), and *B.neuwiedi* and *Crotalusdurissus* (*n* = 1; 9% each).

**Table 1.** List of species found in the Parque Estadual da Serra do Papagaio, Minas Gerais, Brazil. Abbreviations: abundance (N), relative frequency of each species (f%), environment (1 - High altitude grassland, 2 - Rocky Field, 3 - Dense Montane Ombrophilous Forest, 4 - Mixed Ombrophilous Forest), habitat (aa - open area, bf - forest edge, da – disturbed areas, fl - forest, lo - lotic environment, le - lentic environment), and habits (F-fossorial, C-cryptozoic, SAQ-sub-aquatic, T-terrestrial, SA-sub-arboreal, and A-arboreal). Species registered outside the park area (*).

Family/ Species	N	F%	Environments	Habitat	Habit	Altitudinal variation (m)	New record altitudinal (m)
COLUBRIDAE							
*Chironiusbicarinatus* (Wied, 1820)	1	1.2	1,3	bf, at	SA	0–1850	–
*Chironiusbrazili* Hamdan & Fernandes, 2015	1	1,2	1,3	Bf, fl, at	SA	200–2030	–
*Spílotes pullatus* (Linnaeus, 1758)	2	2.5	3	fl	SA	0–1100	1630
DIPSADIDAE							
*Apostolepisassimilis* (Reinhardt, 1861)*	1	1.2	1	Aa, at	F, T	170–1610	–
*Atractuszebrinus* (Jan, 1962)	15	18.7	1,2,3	Aa, fl, at	F, T	20–1610	1730
*Boirunamaculata* (Boulenger, 1896)	1	1.2	3	fl	T	0–1880	–
*Echinantheracephalostriata* Di-Bernardo, 1996	2	2.5	3	Bf, fl	C, T	0–1610	1730
*Erythrolamprusmiliaris* (Linnaeus, 1758)	1	1.2	3	Fl, le	SAQ	–	1643
*Gomesophisbrasiliensis* (Gomes, 1918)	8	10	1	Aa, lo, le, at	SAQ	430–1650	1750
*Mussuranamontana* (Franco, Marques & Puorto, 1997)*	1	1.2	4	fl	T	750–1610	1740
*Oxyrhopusclathratus* Duméril, Bibron & Duméril, 1854*	1	1.2	1	Aa, at	T	0–1610	–
*Oxyrhopusrhombifer* Duméril, Bibron & Duméril, 1854	1	1.2	1	Aa, at	T	0–1330	1730
*Philodryasaestiva* (Duméril, Bibron & Duméril, 1854)	2	2.5	1,2	aa	T	0–1730	1800
*Philodryaspatagoniensis* (Girard, 1858)	9	11.2	1,2	Aa, at	T	0–1600	2200
*Sibynomorphusmikanii* (Schlegel, 1837)	1	1.2	1	Aa, at	T	110–1350	1630
*Taeniophallusaffinis* (Günther, 1858)	3	3.7	1,3	aa	T, C	0–1600	1760
Taeniophallusgr.occipitalis*	1	1.2	1	bf	T, C	–	1600
*Thamnodynastesstrigatus* (Günther, 1858)	15	18.7	1,3,4	Aa, fl, bf, at	SAQ	0–2450	–
*Tomodondorsatus* Duméril, Bibron & Duméril, 1854	2	2.5	3,4	fl	T	0–1610	1730
*Xenodonmerremii* (Wagler *in* Spix, 1824)	1	1.2	1,2	aa	T	0–1300	1610
VIPERIDAE							
*Bothropsfonsecai* Hoge & Belluomini, 1959	7	8.7	1,2,3,4	Aa, bf, fl	T	440–1730	2175
*Bothropsjararaca* (Wied, 1824)	2	2.5	1,2,3,4	Bf, fl	T	0–1640	2150
*Bothropsneuwiedi* Wagler *in* Spix, 1824	1	1.2	1,2	Aa, bf	T	0–1600	2150
*Crotalusdurissus* Linnaeus, 1758	1	1.2	1,2	Aa, bf	T	0–1400	1950
Total	80	100					

### Comparison with other snake assemblages from the Atlantic Forest of southeastern Brazil

**Figure 2.**
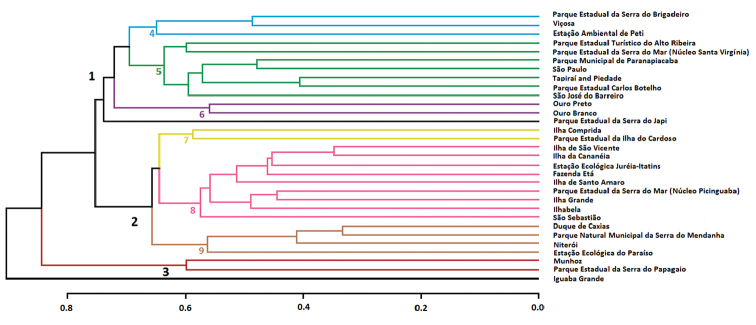
Cluster analysis based on snake species composition from 31 localities of the Atlantic Forest, southeastern Brazil.

The cluster analysis (cophenetic correlation coefficient = 0.8381) based on 120 snake species recorded at 31 localities, including Parque Estadual da Serra do Papagaio, resulted in three main groupings (Fig. 2).

Group 1 was composed generally by localities of mid to high altitudes (> 600 m): the coastal forests of Serra do Mar and Bahia Interior Forests (Olson et al. 2001), covering the Paranapiacaba, Cantareira and Bocaina Mountains in São Paulo, and the Mantiqueira and extreme south of Espinhaço mountains in Minas Gerais. An exception was the Parque Estadual da Serra do Japi, which has remained isolated from the other localities. Three subgroups can be visualized in this grouping. The first (4) is formed by areas in the northern portion of Serra da Mantiqueira and surroundings (Parque Estadual da Serra do Brigadeiro and Viçosa), in Minas Gerais state, as well as a locality in southern Espinhaço Mountains (Estação Ambiental de Peti). The second (5) is comprised of the mountains in São Paulo State. The third (6) is composed of two localities in the southern region of Serra do Espinhaço (Municipalities of Ouro Branco and Ouro Preto and surroundings).

Group 2 consisted of low areas (< 400 m a.s.l.) of the Coastal Forests of Serra do Mar, comprising island and continental regions in the coastal strip of São Paulo and Rio de Janeiro states. In this group, three subgroups can also be observed. The first (7) consists of two continental islands on the south coast of São Paulo state (Ilha do Cardoso and Ilha Comprida). The second (8) composed by the other insular and lowland locations of the Atlantic Forest in São Paulo state, in addition to an island in Rio de Janeiro State (Ilha Grande). Geographically, this subgroup is close to the Parque Estadual da Serra do Mar (Picinguaba, São Paulo State), with which it shares several snake species. The third (9) is composed of lowland locations in the state of Rio de Janeiro, east of Serra dos Órgãos, for which similarities have already been described by Citeli et al. (2016).

Group 3 is composed of only two high altitude localities (> 1100 m a.s.l.) in the southern Mantiqueira Mountains: the Parque Estadual da Serra do Papagaio and Munhoz.

**Figure 3.**
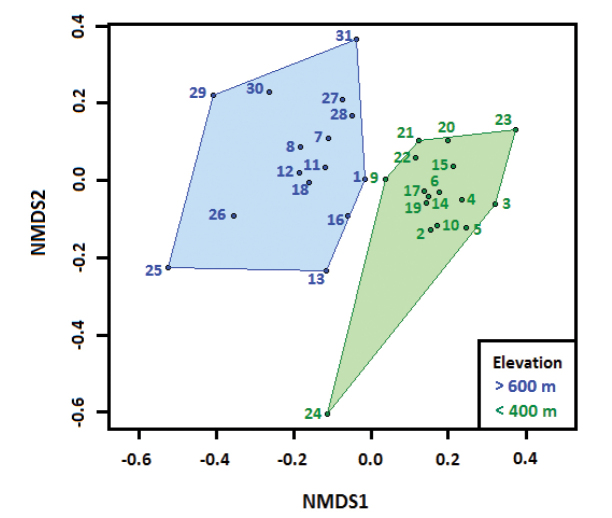
Groupings formed through NMDS analysis (stress 0.1547). The relationship between altitude and the composition of snake species in the Atlantic Forest of southeastern Brazil is shown. The numbers correspond to the same localities as in figure 1.

The NMDS ordering analysis (stress 0.1547) graphically depicts the relative position of localities in a two-dimensional space (Fig. 3). It suggests the clustering pattern found between the locations is associated with the spatial distribution, mainly altitudinal, in the study area and corroborates the results of the cluster analysis.

### Natural history information about the species

#### Colubridae Oppel, 1811

##### Chironius bicarinatus

Taxon classificationAnimaliaSquamataColubridae

(Wied, 1820)

0D8CBAAC-CD35-5A49-9D4B-5E2EEC89A447

###### Natural history notes.

Medium-sized snake (*n* = 1), diurnal and semi-arboreal (Marques et al. 2001). An individual was observed on the ground during the day (12:00 h) in March, next to a small fragment of disturbed forest. Upon detecting the observer’s approach, the snake fled into the forest. Sazima and Haddad (1992) also mention the presence of *C.bicarinatus* in fragments of disturbed forests. The diet is specialized in anurans, composed mainly of hylids and leptodactylids (Dixon et al. 1993). Reproduction is seasonal, with copulation in early autumn and between 4 – 14 eggs laid at the end of winter (Marques et al. 2009, Pontes and Rocha 2008).

###### Altitudinal variation.

From sea level, from the northern coast of Rio Grande do Sul to Bahia, to a maximum altitude of 1610 m in Campos do Jordão, SP (Bérnils 2009). In this study, the maximum altitudinal record was 1730 m, in Baependi, MG. Dixon et al. (1993) cited the species in “*Brazil, Rio de Janeiro, Marombe* [sic], *Itatiaia*,” at 1850 m a.s.l.. The Maromba region encompasses altitudes from 500 to 2000 m. Despite several records of this species in elevated areas (above 800 m a.s.l.) (Bérnils 2009), *Chironiusbicarinatus* is thought to occupy predominantly plains (Dixon et al. 1993, Carreira et al. 2005).

###### Distribution and habitat.

Northeast, central-west, southeast and south of Brazil (Bahia, Goiás, Mato Grosso do Sul, Minas Gerais, Espírito Santo, Rio de Janeiro, São Paulo, Paraná, Santa Catarina and Rio Grande do Sul), Argentina, and Uruguay (Bérnils 2009, Wallach et al. 2014). This species inhabits all forest formations and open areas such as pampas, cerrado, restingas (Sazima and Haddad 1992, Dixon et al. 1993, Carreira et al. 2005) and rocky fields.

##### Chironius brazili

Taxon classificationAnimaliaSquamataColubridae

Hamdan & Fernandes, 2015

D96C0A19-0ABC-537B-9B86-63B35D13AB83

Figure 4A

###### Natural history notes.

Medium-size species (*n* = 1), diurnal and semi-arboreal (Dixon et al. 1993, Marques et al 2015). Five observations of *C.brazili* were made (one during fieldwork and four outside the sampling period). Three individuals were observed between the stones of a waterfall (10:00 – 15:00 h). A recently road-killed adult male was found during the day (11:00 h) in a forest area. Additionally, we observed an individual at rest, coiled over the vegetation at 1.30 m above the ground during the day (16:30 h). All records occurred near watercourses. Abegg et al. (2016) also mention the occurrence of *C.brazili* in riparian forests. No data on the diet was obtained from the examined specimen. However, as in the other species of the genus, it is likely that *C.brazili* preys primarily on anurans, mainly hylids (Dixon et al. 1993). We could observe the following defensive behaviors for *C.brazili*: head elevation and neck S-coil.

###### Altitudinal variation.

The maximum altitudinal record for the species was at 2030 m a.s.l. at Pico do Inficionado, Catas Altas, MG (Bérnils 2009). In the present study, the maximum altitudinal record was at 1600 m a.s.l., in Baependi, MG.

###### Distribution and habitat.

Central-west, southeast and south of Brazil (Federal District, Goiás, Minas Gerais, São Paulo and Rio Grande do Sul) (Hamdan and Fernandes 2015). This species is thought to live in habitats similar to those of *C.flavolineatus* and inhabits riverine forests and forest borders near open areas (Hamdan and Fernandes 2015).

**Figure 4.**
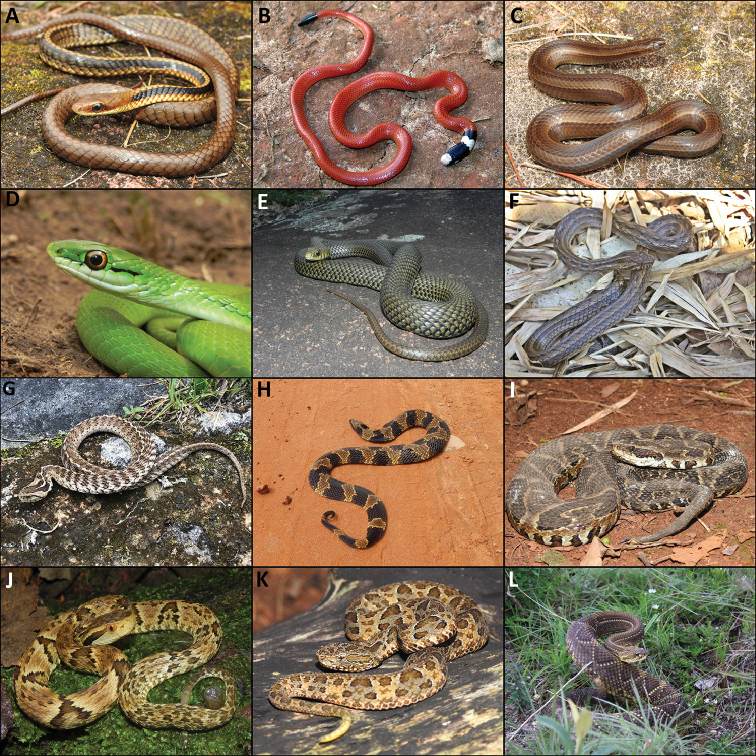
Snakes from the Serra do Papagaio. **A***Chironiusbrazili***B***Apostolepisassimilis***C***Gomesophisbrasiliensis***D***Philodryasaestiva***E***Philodryaspatagoniensis***F***Thamnodynastesstrigatus***G***Tomodondorsatus***H***Xenodonmerremii***I***Bothropsfonsecai***J***Bothropsjararaca***K***Bothropsneuwiedi***L***Crotalusdurissus*. Photographs by Mário Sacramento, Frederico de Alcântara Menezes, Arthur Diesel Abegg, and Leonardo Chaves.

##### Spilotes pullatus

Taxon classificationAnimaliaSquamataColubridae

(Linnaeus, 1758)

BB9F9CC3-61BA-5794-89E7-77E4F5B3A2EC

###### Natural history notes.

A large species (*n* = 1), with semi-arboreal habits, and diurnal activity (Marques et al. 2001, Marques and Sazima 2004). Both of our records were made in the same place, during the day (12:00 and 12:10 h), in September and October, respectively, indicating that it may be the same individual. On both occasions, the individuals were on the ground, in a forest area. The diet is mainly composed of mammals (Marques et al. 2014), but *S.pullatus* also feeds on lizards, birds and their eggs, and anurans (Martins and Oliveira 1998, Marques and Sazima 2004, Bernarde and Abe 2006). This species lays five to twelve eggs (Amaral 1930, Marques et al. 2014). No defensive behavior was observed for this species.

###### Altitudinal variation.

In Brazil, range spans at minimum of sea level from the coast of Santa Catarina to Bahia to a maximum of 1100 m a.s.l., Brasília, Federal District (Bérnils 2009). The maximum altitudinal record of this species for Brazil is expanded here by our observations at 1630 m a.s.l.. From the toponyms surveyed, Bérnils (2009) recorded only 8% in areas higher than 800 m a.s.l. and 57% between the range of sea level and 400 m a.s.l.

###### Distribution and habitat.

This species can be found in all Brazilian states, and in Argentina and Paraguay (Wallach et al. 2014). It lives in open formations associated with riparian, dense and seasonal ombrophilous forests (Bérnils 2009).

#### Dipsadidae Bonaparte, 1838

##### Apostolepis assimilis

Taxon classificationAnimaliaSquamataDipsadidae

(Reinhardt, 1861)

74709F12-A0F6-5B1E-9628-E639A348F1FA

Figure 4B

###### Natural history notes.

Species of small size (*n* = 1), with nocturnal activity and cryptozoic or fossorial habits (Ferrarezzi et al. 2005, Marques et al. 2015). In December, an adult was seen at 07:30 h. moving in an open area. The record occurred in Aiuruoca-MG, near the PESP. The diet is composed of amphisbaenids and other elongate vertebrates (Ferrarezzi et al. 2005). Barbo (2011) mentions two females with four and six vitellogenic follicles in November and March. No defensive behavior was observed for this species.

###### Altitudinal variation.

This species was found at a minimum of 170 m a.s.l., in Cuiabá, Mato Grosso and maximum of 1610 m a.s.l. in Campos do Jordão, SP (Bérnils 2009). In this study, the record occurred outside the park limits at 1100 m a.s.l., in Aiuruoca, MG.

###### Distribution and habitat.

Northeast, central-west, southeast and southern Brazil (Bahia, Mato Grosso, Mato Grosso do Sul, Goiás, Distrito Federal, Minas Gerais, São Paulo and Paraná), southeast Paraguay, northern Argentina, and Bolivia (Bérnils 2009; Wallach et al. 2014). This species is typical of the Cerrado morphoclimatic domain, also occurring in adjacent forested areas (Ferrarezzi et al. 2005).

##### Atractus zebrinus

Taxon classificationAnimaliaSquamataDipsadidae

(Jan, 1862)

A9FAA999-C31E-52E8-B697-9F9C6B47424B

###### Natural history notes.

Species of small size (*n* = 15), nocturnal, with fossorial habits (Marques et al. 2001). Data on *A.zebrinu* activity and habitat use are available from sparse records and inferred from ecological attributes of other species of the genus (e.g., *Atractuspantostictus* and *A.reticulatus*). The species was considered abundant in the study area and was found in all the sampled vegetation types. The exception was mixed ombrophilous forest, which are located in valleys along water courses and undergo seasonal flooding in the rainy season. This species was often captured in pitfall traps (open area = 7, forest area = 8, pitfall = 8). Seven individuals were found during the day (09:30–18:00 h.), all at rest; three under trunks, three were buried and came up when a tractor revolved the soil, and one was basking. An adult female was observed at 20:00 h. crossing the road in a forest area. This information reinforces the conclusion that this species is nocturnal and a generalist for vegetation use, because it was found in both open and forested areas. Similar to other *Atractus* species, the diet is composed of annelids (Marques et al. 2001). Of the seven individuals examined, two had earthworms in their stomachs. This species was found in all sampling months at similar numbers. There is no information in the literature regarding reproduction. A female collected in October (SVL = 425 mm; TL = 34 mm) presented four vitellogenic follicles and another female was found in December (SVL = 480 mm; TL = 40 mm) with 12 undeveloped follicles. In June, two young individuals (approx SVL = 200 mm) were found together when a tractor revolved the earth. Passos et al. (2016) observed aggregation of juveniles after parturition in *Atractuspotschi*. As defensive tactics, we observed cloacal discharge and head hiding.

###### Altitudinal variation.

Found at a minimum of 20 m a.s.l. in Itaboraí, RJ and maximum of 1610 m a.s.l. in Campos do Jordão, SP (Bérnils 2009). The maximum altitude of the species is expanded here by individuals we observed at 1730 m a.s.l.

###### Distribution and habitat.

South and southeast of Brazil (Santa Catarina, Paraná, São Paulo, Rio de Janeiro, Minas Gerais and Espírito Santo) (Bérnils 2009, Wallach et al. 2014). The toponyms occupied by this species are concentrated in the coastal mountain ranges of Paraná and São Paulo States: Paranapiacaba, Órgaos, Mantiqueira, and Espinhaço mountain ranges. The species is found in areas with predominantly dense ombrophilous forests, mixed ombrophilous forest, seasonal forest (Bérnils 2009), and high altitude grasslands.

##### Boiruna maculata

Taxon classificationAnimaliaSquamataDipsadidae

(Boulenger, 1896)

DB195237-CF9C-5204-AAD9-E4A809957BCE

###### Natural history notes.

A large species (*n* = 1), terrestrial (Marques et al. 2015), although there is one record of arboreal substrate (Gallardo et al. 2006). In October, a juvenile was collected at 07:30 h. while crossing an unpaved road in a forested area. Data on daily activity are scarce. In the literature, there are two observations of activity at night, one during twilight, and one during the day (Gallardo et al. 2006, Hartmann and Giasson 2008, Gaiarsa et al. 2013). Although the specimen was found active in the early hours of the day, it is thought that the species is predominantly nocturnal, similar to other Pseudoboine species (Marques 1998, Sawaya et al. 2008). A recently ingested lizard (*Ophiodes* sp.), swallowed head-first, was found in the digestive tract of this specimen. Previous studies indicate this species is a generalist, feeding primarily on snakes, but also birds, small mammals, anurans, and lizards (Lema et al. 1983, Carreira 2002, Gallardo et al. 2006, Hartmann and Giasson 2008, Gaiarsa et al. 2013). This is the first record of an *Ophiodes* as prey for *B.maculata*. No reproductive data were obtained from the examined specimen. However, the species is known to lay from four to 15 eggs (Sawaya et al. 2008). No defensive behavior was observed for this species.

###### Altitudinal variation.

Species records indicate a minimum of sea level in Rio Grande do Sul and maximum of 1240 m a.s.l. in Serra do Salitre, MG (Bérnils 2009). In Brazil, the maximum altitudinal record of the species is for the study area (at 1600 m a.s.l.). Observations at toponyms below 400 m a.s.l. were recorded only on the coast, from Uruguay to Rio Grande do Sul, and in the western and southernmost parts of its distribution (Negro, Jacuí, Uruguay, Paraguay, and Paraná basins) (Bérnils 2009). Quinteros-Muñoz (2006) collected an individual at 1880 m a.s.l. in the National Park La Yunga, Bolivia in a region that encompasses altitudes ranging from 1000 to 4000 m a.s.l.

###### Distribution and habitat.

North, central-west, southeast and south of Brazil (Amazonas, Distrito Federal, Goiás, Mato Grosso do Sul, Minas Gerais, Rio Grande do Sul, São Paulo), Argentina, Paraguay, and Uruguay (Bérnils 2009, Wallach et al. 2014). Typically, this is a species of open areas (cerrados, savannas, chacos, and pampas) with records near adjacent forests (Bérnils et al. 2007).

##### Echinanthera cephalostriata

Taxon classificationAnimaliaSquamataDipsadidae

Di-Bernardo, 1996

CE558160-9BF1-5FAC-92EA-EC2FF3B648A0

###### Natural history notes.

Species of small size (*n* = 2), predominantly diurnal, terrestrial and cryptozoic (Marques et al. 2001). Feeds on anurans (Marques et al. 2001, Forlani et al. 2010). In September, a female was found in the early afternoon (12:00 h), inside a bromeliad (*Aechmeaaiuruocensis* Leme) on the forest floor. In its digestive tract, we found a *Physalaemusolfersii* (Lichtenstein & Martens, 1856) (ingested by the leg), and three anuran eggs. Moura-Leite et al. (2003) recorded a specimen with 32 anuran eggs in the stomach. The second individual, another female, was found resting during the day. The record was made in November, at the forest edge, near a swamp area. This individual had ingested three *Physalaemusjordanensis* Bokermann, 1967 (two were ingested head-first and the last by the leg). Pontes et al. (2008) found *E.cephalostriata* only in primary and secondary forests. The presence of *P.jordanensis* (found in PESP just in open area marshes, F. Menezes pers. obs.) as prey of *E.cephalostriata*, indicates this snake also forages in open areas and lentic environments. Reproduction data of the species are scarce. The female collected in September (SVL = 490 mm; TL = 210 mm) presented nine vitellogenic follicles. Fiorillo (2016) refers to a female from Iguape, with eight vitellogenic follicles in November. No defensive behavior was observed for this species.

###### Altitudinal variation.

Found at altitude minimum of sea level from Santa Catarina coast to Rio de Janeiro and maximum of 1610 m a.s.l. in Campos do Jordão, SP (Bérnils 2009). The maximum altitude for this species is recorded here, with two individuals found at 1730 m a.s.l.. Bérnils (2009) mentioned that more than 80% of the toponyms come from hill and plateau areas. Although the distribution range is concentrated in mountains and plateau areas, this species is also frequent in lower altitudes (see Hartmann 2005, Fiorillo 2016).

###### Distribution and habitat.

Northeast, southeast, and southern Brazil (Bahia, Espírito Santo, Minas Gerais, Paraná, Rio de Janeiro, Santa Catarina and São Paulo) (Wallach et al. 2014). Occurs in dense and mixed ombrophilous forest formations, as well as in semi-deciduous forests (Di-Bernardo 1996, Bérnils 2009).

##### Erythrolamprus miliaris

Taxon classificationAnimaliaSquamataDipsadidae

(Linnaeus, 1758)

D5720AC6-FCF3-5FB9-A4DB-F535C75BFC85

###### Natural history notes.

Species of medium size (*n* = 1), predominantly diurnal and semi-aquatic (Marques et al. 2001), although there are reports of activity at night (Sazima and Haddad 1992). In September, an individual was found standing still on the forest floor during the day (15:00 h) at a swamp border. This species feeds mainly on amphibians, fish, and tadpoles (Marques and Souza 1993, Marques and Sazima 2004), although it can occasionally feed on other reptiles (Marques and Sazima 2004, Bonfiglio and Lema 2007, Hartmann et al. 2009a). Pizzatto and Marques (2006) reported a continuous reproductive cycle in a population of southern Bahia state coast (northern distribution) and seasonal reproductive cycles in populations of both inland and coastal São Paulo and Paraná states (southern distribution). Fecundity is six to seventeen eggs, with individuals reaching sexual maturity at twelve months (Vitt 1992, Pizzatto and Marques 2006). No defensive behavior was observed for this species.

###### Altitudinal variation.

We found no information in the literature regarding the altitudinal variation of the species. In this study, the maximum altitudinal record was at 1643 m a.s.l., in Baependi-MG.

###### Distribution and habitat.

Northern, northeast, central-west, southeast and southern Brazil (Alagoas, Amapá, Amazonas, Bahia, Ceará, Espírito Santo, Goiás, Maranhão, Mato Grosso, Mato Grosso do Sul, Minas Gerais, Pará, Paraná, Pernambuco, Piauí, Rio de Janeiro, Rio Grande do Norte, Rio Grande Do Sul, Rondônia, Santa Catarina, São Paulo, Sergipe) (Wallach et al. 2014). This species occurs in semi-deciduous, dense, and mixed ombrophilous forests, as well as in adjacent open areas, from the Amazon to the Atlantic Forest (Gans 1964).

##### Gomesophis brasiliensis

Taxon classificationAnimaliaSquamataDipsadidae

(Gomes, 1918)

B33C87BE-F5CF-5FF4-8801-9FC7DD3A6DEF

Figure 4C

###### Natural history notes.

Species of small size (*n* = 8). This species is considered nocturnal and aquatic, associating with lentic watercourses (Gomes 1918, Marques et al. 2001, Gonzalez et al. 2014). During our fieldwork, it was found during all months of sampling in similar numbers and all observations occurred during the day in open areas. Five individuals were found active: four were moving from a river edge towards a swamp (10:00 h, 16:00 h, 17:00 h, 17:30 h) and one towards a creek (14:00 h). Three individuals were found at rest; one on a creek edge (9:00 h.), one in a muddy area (15:00 h) and another in a swamp (16:00 h). Fortes et al. (2010) recorded two active individuals at 21:00 h swimming on the surface of an 80-cm-deep turbid water lagoon. A male (SVL = 420 mm; TL = 75 mm) was kept in a 70cm × 30cm × 45cm terrarium for 10 consecutive days. During this period, it was monitored by camera 24 hours/day, to study its activity. It presented a unimodal activity pattern, with 96.4% of activity records during the day and peak activity from 9:30 to 17:00 h. Data obtained in the laboratory and field observations indicate this species is predominantly diurnal and semi-aquatic. *G.brasiliensis* frequently uses the ground (instead of the water), mainly during the day, to move between lentic and lotic environments. Of the three specimens examined, one had an earthworm in its stomach. Our results are consistent with the study by Oliveira et al. (2003) who also found traces of earthworm in the digestive tract of *G.brasiliensis*. Earthworms are sensitive to light and ultraviolet radiation (Edwards and Lofty 1977), so they are predominantly nocturnal, coming to the surface at night or during periods of very low light intensity during the day (Lee 1985). The diurnal activity of *G.brasiliensis* does not match its prey activity period. *G.brasiliensis* may hunt and capture its prey underground, during the day, possibly on the borders of marshes where the concentration of earthworms is higher (Frederico Menezes, pers. obs.). No information about reproduction of the examined specimens was recorded, except for a pregnant female in February. The species has seasonal reproduction associated with the rainy season, with juvenile recruitment between February and March (Oliveira et al. 2003). The defensive repertoire is described in Menezes et al. (2017).

###### Altitudinal variation.

Found at a minimum of 430 m a.s.l. in Encruzilhada do Sul, RS and maximum of 1650 m a.s.l. in the Parque Estadual da Serra do Papagaio, Alagoa, MG. The maximum altitudinal record derives from the same are in this study, where most individuals were recorded at 1750 m altitude, in the PESP, Baependi, MG. The toponyms obtained for this species occur in two altitudinal ranges: 51% are located between 430 and 800 meters and 49% above this range (Bérnils 2009).

###### Distribution and habitat.

This species occurs in natural field areas (Amaral 1977, Ghizoni-Jr et al. 2009) in southern and southeastern Brazil (Minas Gerais, Paraná, Rio Grande do Sul, Santa Catarina, and São Paulo) (Gonzalez et al. 2014).

##### Mussurana montana

Taxon classificationAnimaliaSquamataDipsadidae

(Franco, Marques & Puorto, 1997)

3DD664B0-487C-55E6-B130-BB5ECC6C1160

###### Natural history notes.

A large species (*n* = 1), terrestrial (Marques et al. 2001, Gaiarsa et al. 2013). There is no information on the time of activity for this species (Gaiarsa et al. 2013). In October, an adult was seen resting at 11:20 h, coiled in the middle of plant litter, in a forested area. The record occurred in an adjacent conservation area: RPP Alto Montana, Itamonte-MG. Literature data indicate its diet is composed of snakes and lizards (Franco et al. 1997). Regarding the reproduction, Franco et al. (1997) recorded two females: one with seven and the other with 11 eggs. No defensive behavior was observed for this species.

###### Altitudinal variation.

This species was found at a minimum of 750 m a.s.l. in Guaratinguetá, SP and maximum of 1610 m a.s.l. in Campos do Jordão, SP (Bérnils 2009). The maximum altitudinal record of the species was 1740 m a.s.l., at RPPN Alto Montana, Itamonte, MG. Bérnils (2009) stated all toponyms found to occur in areas above 750 m a.s.l..

###### Distribution and habitat.

Southeast Brazil (Minas Gerais, Rio de Janeiro, and São Paulo) (Wallach et al. 2014). This species occurs in fields, close to ombrophilous and seasonal forests (Bérnils 2009).

##### Oxyrhopus clathratus

Taxon classificationAnimaliaSquamataDipsadidae

Duméril, Bibron & Duméril, 1854

F065370C-24BD-5386-81BF-9B3D2B070250

###### Natural history notes.

Species of medium size (*n* = 1), terrestrial and nocturnal (Marques et al. 2001). A recently road-killed adult male was found in an open area near the PESP, during the morning. There was no evidence of diet or reproduction of the examined specimen. The available information in the literature indicates a diet composed mainly of mammals, although lizards and birds can also be prey (Hartmann et al. 2009b, Gaiarsa et al. 2013). Reproduction is seasonal, with a reproductive peak in the summer (Marques and Sazima 2004). Fecundity varies from four to 16 eggs (Gaiarsa et al. 2013). No defensive behavior was observed for this species.

###### Altitudinal variation.

The species is found at a minimum of sea level from the coast of Rio Grande do Sul to Rio de Janeiro, and a maximum of 1610 m a.s.l. in Campos do Jordão, SP (Bérnils 2009). In this study, we found this species outside the PESP limits, at 1000 m a.s.l. in the Aiuruoca, MG. Total altitudinal range is broad with 31.7% of toponyms found from the sea level up to 400 m a.s.l., 36.6% between 401 and 800 m a.s.l. and 31.7% above 801 m a.s.l. (Bérnils 2009).

###### Distribution and habitat.

Northeast and southeast Brazil (Bahia, Espírito Santo, Minas Gerais, Rio de Janeiro, Rio Grande do Sul, Santa Catarina, São Paulo), and Argentina (Wallach et al. 2014, Bernardo et al. 2012). It occurs in dense, mixed ombrophilous and seasonal semidecidual forests (Bernardo et al. 2012).

##### Oxyrhopus rhombifer

Taxon classificationAnimaliaSquamataDipsadidae

Duméril, Bibron & Duméril, 1854

2EA88614-498D-51F6-A035-D056B30418F9

###### Natural history notes.

Species of medium size (*n* = 1), nocturnal and terrestrial (Marques et al. 2001). An individual was found resting during the day (9:30 h) in a pasture area. This species occurs mainly in open areas (Cechin 1999, Sawaya et al. 2008), but may be found in forested areas near fields (Lema 1994, Cardoso 2011). It is a generalist species, feeding mainly on lizards, but also small mammals and snakes (Cechin 1999, Carreira 2002, Maschio et al. 2003, Sawaya et al. 2008). It is oviparous, with litter varying from two to 12 eggs (Gaiarsa et al. 2013). No defensive behavior was observed for this species.

###### Altitudinal variation.

This species is found at a minimum of sea level in Argentina, Uruguay, Rio Grande do Sul, and south of Santa Catarina, and at a maximum of 1330 m a.s.l. in Liberdade, MG (Bérnils 2009). This study provides a new maximum altitudinal record of the species with an individual found at 1730 m a.s.l., Baependi, MG. The records located below 400 m a.s.l. occurred only from the Prata Basin to Santa Catarina (Bérnils 2009).

###### Distribution and habitat.

Northeast, central-west, southeast and southern Brazil (Bahia, Ceará, Distrito Federal, Goiás, Mato Grosso, Mato Grosso do Sul, Minas Gerais, Pará, Paraná, Pernambuco, Rio de Janeiro, Rio Grande do Sul, Rondônia, Santa Catarina and São Paulo), Argentina, Bolivia, Paraguay and Uruguay (Wallach et al. 2014). The species was predominantly found in areas with open formations of Pampa, plateau fields, rupestrian fields, restingas, and at the southern portions of the cerrado (Bérnils 2009, Ghizoni-Jr et al. 2009).

##### Philodryas aestiva

Taxon classificationAnimaliaSquamataDipsadidae

(Duméril, Bibron & Duméril, 1854)

00578F1D-616A-550A-A9DC-CBA66D8847B4

Figure 4D

###### Natural history notes.

Species of medium size (*n* = 2), diurnal, often found on the ground in open areas (Di-Bernardo 1998, Marques et al. 2001, Sawaya et al. 2008). A recently road-killed adult male was found in an open field during the morning. The other record is of an adult’s shed skin, located at the grassland, in a rock outcrop, at 1800 m a.s.l.. There is no information on diet and reproduction of the examined specimen. Machado-Filho (2015) describes this species as generalist, feeding on mammals (40%), birds (25%), lizards (20%) and anurans (15%). Vitellogenesis occurs between April and December and ovulation between July and December (Fowler et al. 1998). There is a record of a captive female with eleven eggs (Fowler et al. 1998). No defensive behavior was observed for this species.

###### Altitudinal variation.

This species was found at a minimum of sea level from the coast of Uruguay to Santa Catarina and maximum of 1730 m a.s.l. in Campos do Jordão, SP (Bérnils 2009). The maximum altitudinal record for this species is presented here, with a specimen recorded at 1800 m a.s.l.. Bérnils (2009) mentioned the species occurs at sea level only from Uruguay to Santa Catarina. All other localities where this species was found are plateau areas.

###### Distribution and habitat.

Northeast, central-west, southeast and southern Brazil (Bahia, Distrito Federal, Goiás, Mato Grosso do Sul, Minas Gerais, Paraná, Rio de Janeiro, Rio Grande do Sul, Santa Catarina, and São Paulo), Argentina, Bolivia, Paraguay and Uruguay (Wallach et al. 2014). This species was predominantly found in open areas of pampas, plateau fields, cerrados, and restingas, with records to adjacent forests (Giraudo and Scrocchi 2002, Bérnils 2009).

##### Philodryas patagoniensis

Taxon classificationAnimaliaSquamataDipsadidae

(Girard, 1858)

74586C83-0EDB-546B-8EBF-FC31B6996A88

Figure 4E

###### Natural history notes.

Species of medium size (*n* = 9), diurnal and terrestrial (Marques et al. 2001, Hartmann and Marques 2005). All observations occurred in open areas during the day. Seven individuals were found between 14:00 and 17:00 h, and two were found in the morning. According to Sazima and Haddad (1992), and Hartmann and Marques (2005), this species is active mainly during the hottest hours of the day. In December, during the day (14:00 h), we found two adults (male and female) about two meters away from each other. Both were coiled at rest and showed evidence of being in the shedding process.

In September, an adult was observed at 14:00 h near a ravine, while it was being attacked by two different birds (*Poospiza* sp. and an unidentified Passeriformes), possibly in defense of a nearby nest. In July, an adult was observed at 15:00 h, while it was ingesting a rodent. Out of the four examined specimens, two presented rodents in their stomach. Machado-Filho (2015) suggest this species is generalist, feeding on anurans (27%), lizards (25.8%), mammals (19.4%), snakes (10.9%), birds (8%), spiders (4%), fish (0.4%) and amphibians (0.4%). *P.patagoniensis* was found during all seasons of the year with higher frequency in December (*n* = 4). There is no information on reproduction of the examined individuals. Previous records indicate reproduction is seasonal, with three to nine eggs, secondary vitellogenesis between August and December, and ovulation between October and December (Fowler et al. 1998, Sawaya et al. 2008). As defensive tactics of this species, we observed cloacal discharge, head elevation, head triangulation, and neck S-coiling.

###### Altitudinal variation.

This species was found at a minimum of sea level from the coast of Argentina to the state of Espírito Santo and maximum of 1660 m a.s.l. in Umuarama, Campos do Jordão, SP (Bérnils 2009). The maximum altitudinal record for this species is from the area in this study, where an individual was recorded at 2200 m a.s.l. altitude, in the Alagoa, MG.

###### Distribution and habitat.

North, northeast, central-west, southeast and southern Brazil (Bahia, Distrito Federal, Goiás, Mato Grosso, Mato Grosso do Sul, Minas Gerais, Pará, Paraná, Rio de Janeiro, Rio Grande do Norte, Rio Grande do Sul, Rondônia, and São Paulo), Argentina, Bolivia, Chile, Paraguay and Uruguay (Wallach et al. 2014). This species can be found in open mountain areas, pampas, plateau fields, chacos, cerrados, restingas and deforested areas (Giraudo and Scrocchi 2002, Bérnils et al. 2007).

##### Sibynomorphus mikanii

Taxon classificationAnimaliaSquamataDipsadidae

(Schlegel, 1837)

3B734F43-9D7E-519F-A0DC-8A4B70ACAFA5

###### Natural history notes.

Species of small size (*n* = 1), nocturnal and terrestrial (Marques et al. 2001). A recently road-killed individual was found in an open area during the day. No diet or reproduction data was recovered for the examined specimen. Available information indicates this species is a specialist in mollusks (Marques et al. 2001). Fecundity varies from three to 10 eggs, which may be laid in communal spawning (Albuquerque and Ferrarezzi 2004, Pizzatto et al. 2008a). No defensive behavior was observed for this species.

###### Altitudinal variation.

This species was found at a minimum of 110 m a.s.l., in Puerto Bemberg, Iguazú, Argentina and maximum of 1350 m a.s.l. in the Serra do Ouro Branco, Ouro Branco, MG (Bérnils 2009). The maximum altitude for the species is expanded in this work, where a record occurred at 1630 m a.s.l., in the Baependi, MG. Total altitudinal range is broad with 12.3% of toponyms found below 400 m a.s.l., 30.5% above 801 m a.s.l., and 57.2% in the range between 401 and 800 m a.s.l. (Bérnils 2009).

###### Distribution and habitat.

Northeast, central-west, southeast and southern Brazil (Bahia, Goiás, Maranhão, Mato Grosso do Sul, Minas Gerais, Pará, Paraná, Norte and São Paulo), Argentina and Paraguay (Bérnils 2009, Wallach et al. 2014). The species is common in forested formations from the Amazon Forest to the Atlantic Forest, in semidecidual and riverine forests and savannah formations of cerrado (Bérnils 2009, Freitas et al. 2014).

##### Taeniophallus affinis

Taxon classificationAnimaliaSquamataDipsadidae

(Günther, 1858)

D209AEE4-C505-52F1-887D-F25D29431578

###### Natural history notes.

Species of small size (*n* = 3), diurnal, terrestrial and cryptozoic (Marques et al. 2001). Two recently road-killed individuals were found during the day at 9:00 h and 14:00 h: one in an open area and the other in a forested area. A third individual was also found during the day in an open area, apparently at rest, near a watercourse. Of the three examined specimens, one presented fragments of anurans in its digestive tract. Available information indicates the diet is composed of anurans primarily, but also by lizards and amphisbaenians (Marques et al. 2001, Barbo and Marques 2003). There is no information in the literature regarding the reproduction of the species. A female (SVL = 397 mm TL = 125 mm) presented five vitellogenic follicles in September. No defensive behavior was observed for this species.

###### Altitudinal variation.

This species is found at a minimum of sea level from the coast of Rio Grande do Sul to Rio de Janeiro and maximum at 1600 m a.s.l. in Parque Estadual Ibitipoca, Lima Duarte, MG (Bérnils 2009). The maximum altitude for the species is increased, where individuals were observed at 1760 m a.s.l., in Baependi, MG. Bérnils (2009) points out that more than 80% of the toponyms are located in mountains and plateaus above 800 m.

###### Distribution and habitat.

Northeast, southeast and southern Brazil (Alagoas, Bahia, Ceará, Espírito Santo, SE Minas Gerais, Paraná, Rio de Janeiro and Rio Grande do Sul, Santa Catarina, São Paulo) (Wallach et al. 2014). This species was reported in areas with predominantly dense and mixed ombrophilous forests (Bérnils 2009).

##### Taeniophallus gr. occipitalis

Taxon classificationAnimaliaSquamataDipsadidae

92474224-3288-54C5-931D-72C2F9A414FC

###### Natural history notes.

Species of small size (*n* = 1), diurnal and cryptozoic (Sawaya et al. 2008). This individual was killed during the day by a domestic cat, near a forested area. There is no information on diet or reproduction for the examined specimen. There are no records of reproduction or altitudinal variation in T.gr.occipitalis. Prior reports are limited on diet, indicating only lizards as prey (Cechin 1999). No defensive behavior was observed for this species.

###### Altitudinal variation.

In this study, the maximum record was at 1600 m a.s.l., in the Aiuruoca, MG.

###### Distribution and habitat.

North, northeast, central-west, southeast and southern Brazil (Bahia, Ceará, Distrito Federal, Goiás, Pará, Paraíba, Paraná, Piauí, Rio Grande do Sul, Rondônia, São Paulo and Sergipe), Argentina, Bolivia, Paraguay, Peru and Uruguay (Wallach et al. 2014, Santos-Jr et al. 2008). Taeniophallusgr.occipitalis occurs in open (cerrados, amazon savannas, plateau fields and pampas) and forested areas (western Amazon Forest and northeastern Atlantic Forest, in Brazil) (Santos-Jr et al. 2008).

##### Thamnodynastes strigatus

Taxon classificationAnimaliaSquamataDipsadidae

(Günther, 1858)

DB7E9E89-EE32-55F3-9268-4997D42A3A0F

Figure 4F

###### Natural history notes.

Species of medium size (*n* = 15), nocturnal and terrestrial (Marques et al. 2001, Barbo et al. 2011). The species was frequently found in November, December, and January. Ten individuals were observed resting during the day between 9:00 and 15:00 h; nine were in open areas and one on a forest border. Three individuals were found active at 22:00 h, foraging on the margin of a marsh with intense anuran vocal activity. Bernarde et al. (2000a) also observed this aggregation in *T.strigatus* in a permanent pond in Parque Estadual da Mata dos Godoy, Londrina, Paraná State. A juvenile was collected at 15:00 h while crossing an unpaved road after heavy rain. An adult was observed, also during the day (9:00 h), as it had captured by the leg and was attempting to prey on a *Leptodactylus* sp. Histological features of the retina of *T.strigatus* (i.e., presence of cones, but absence of rods) (Hauzman et al. 2014) along with activity data obtained in captivity (Torello-Vieira and Marques 2017) reinforce the idea this snake exhibits significant activity during the day. Of the seven individuals examined, three presented stomach contents: lizard scales (in a young individual), a *Physalaemus* sp., and a *Rhinella* sp. (this last one also showed traces of an unidentified exoskeleton - possibly a secondary prey). Bernarde et al. (2000b) suggested *T.strigatus* is a generalist, feeding primarily on anurans (71.4%), but also rodents (14.3%), fish (3.6%), and lizards (3.6%). In regard to reproduction, one female (SVL = 585 mm; TL = 155 mm, collected in December) possessed 14 vitellogenic follicles and a young individual (SVL = 200mm; TL = 65 mm) was recorded in January. Barbo et al. (2011) mentioned observations of two females: one with 15 vitellogenic follicles in February and another one in November with 24 embryos. We could observe the following defensive behaviors for this species: cloacal discharge, head triangulation, body flattening, strike, and biting.

###### Altitudinal variation.

This species was found at a minimum of sea level and maximum of 2450 a.s.l. in Itatiaia National Park, state of Rio de Janeiro, Brazil (Winkler et al. 2011). In this study, the maximum altitudinal record of the species was at 1730 m a.s.l., in the Baependi, MG.

###### Distribution and environment.

Southern, southeast, and northern Brazil (Espírito Santo, Minas Gerais, Pará, Paraná, Rio de Janeiro, Santa Catarina, Rio Grande do Sul, Roraima and São Paulo), Paraguay, Uruguay and Argentina (Franco and Ferreira 2003).

##### Tomodon dorsatus

Taxon classificationAnimaliaSquamataDipsadidae

Duméril, Bibron & Duméril, 1854

A542CA2B-ECC0-5081-AD76-BB783FBE5E78

Figure 4G

###### Natural history notes.

Species of medium size (*n* = 2), diurnal and terrestrial (Marques et al. 2001). Two individuals were found in forested areas during the day, one active at 12:30 h and the other a recently road-kill found at 10:30 h. These records were observed in September and October. We found no data on diet or reproduction of the examined specimens. Prior literature accounts suggest feeding exclusively on slugs. The reproductive cycle is seasonal, with births occurring from January to June (Bizerra et al. 2005). We observed the following defensive behaviors for *T.dorsatus*: cloacal discharge, head triangulation, body flattening, strike and biting.

###### Altitudinal variation.

This species is found at a minimum of sea level from the coast of Rio Grande do Sul to Rio de Janeiro, and a maximum of 1610 m a.s.l. in Campos do Jordão, SP (Bérnils 2009). We contribute a new maximum altitudinal record for our study area, where an individual was observed at 1730 m a.s.l., in Baependi, MG.

###### Distribution and habitat.

Central-west, southeast and southern Brazil (Minas Gerais, Paraná, Rio Grande do Sul, Santa Catarina, and São Paulo), Argentina, Paraguay and Uruguay (Bérnils 2009, Wallach et al. 2014). This species is common in the Atlantic forest areas, with some records to open adjacent areas (Bérnils 2009).

##### Xenodon merremii

Taxon classificationAnimaliaSquamataDipsadidae

(Wagler, 1824)

A303DFDA-FBAA-59F0-963F-727178B9402F

Figure 4H

###### Natural history notes.

Species of medium size (*n* = 1), diurnal and terrestrial (Marques et al. 2001). In January, an individual was found at 14:00 h crossing an unpaved road in an open area. Prior records indicate this species specialize in anurans, mainly the toxic *Rhinella* spp. (Vitt and Vangilder 1983, Jordão 1997). *X.merremii* has a long reproductive cycle, from the beginning of the dry season to the middle of the rainy season. Fecundity varies between six and 44 eggs with recruitment between January and May (Pizzatto et al. 2008). As a defensive tactic of *X.merremii*, we observed the following behavior: body flattening.

###### Altitudinal variation.

This species is found at a minimum of sea level from the northern coast of Rio Grande do Sul to the extreme south of Santa Catarina, São Paulo, Rio de Janeiro, Espírito Santo, and Bahia. Maximum altitude recorded is 1300 m a.s.l. in the Parque Estadual de Itacolomi, Ouro Preto, MG (Bérnils 2009). This study expands the maximum altitudinal record for this species with an individual registered at 1610 m a.s.l. from the Aiuruoca, MG. This species occupies a large variety of habitats, both vegetal and altitudinal. The recorded toponyms corresponds to the altitudinal gradient with 35.3% of toponyms between sea level and 400 m a.s.l., 33.2% between 401 and 800 m a.s.l. and 31.5% above 801 m a.s.l. (Bérnils 2009).

###### Distribution and habitat.

North, northeast, central-west, southeast, and southern Brazil (Bahia, Brasília, Ceará, Goiás, Mato Grosso, Pará, Paraiba, Paraná, Pernambuco, Rondônia, São Paulo, and Tocantins), Bolivia and Paraguay (Wallach et al. 2014). This species occurs mainly in open areas (e.g., Cerrado, Chaco, plateau fields, rocky fields and Caatinga), but is also present in arboreal formations, such as seasonal forests, secondary forests, riverine forests and restingas (Bérnils 2009).

#### Viperidae Oppel, 1811

##### Bothrops fonsecai

Taxon classificationAnimaliaSquamataViperidae

Hoge & Belluomini, 1959

11AF9127-5AE3-5B6F-8065-456D985CD825

Figure 4 I

###### Natural history notes.

Species of medium size (*n* = 7), nocturnal and terrestrial (Marques et al. 2001). We collected twelve specimens of *B.fonsecai*, (seven during fieldwork and five outside of designated fieldwork periods. Individuals were more frequently observed in February and March. All observations occurred during the day. Ten adults were observed. Seven were at rest, five in open areas (at 9:00, 9:30, 10:00, 14:00, 14:10 h) and two at a forest edge (9:00 and 11:30 h). Three were found moving, two in open areas (10:00 and 14:00 h) and one entering a forested area (14:50 h). In November, we found an adult female, at rest at 9:00 h, 50 m away from a forested area. This female was about to shed. We found the same individual again at 14:00 h at the same place, with the skin-shed next to it. At 17:00 h, it had already retreated under the bush (goat’s beard), remaining coiled in a stalking position. All individuals found in open areas were at most 100 m from a forested area. Two juveniles were found in forested areas, one coiled on the ground in the light-shade mosaics made by the sunlight (12:40 h) and another stretched over the first branches of a bromeliad (*Vrieseasceptrum* Mez)(9:30 h). This individual (SVL = 263 mm; TL = 38 mm; M = 18 g) was collected and contained a freshly ingested rodent (M = 6 g). *B.fonsecai* preys exclusively on rodents (Martins et al. 2001). In PESP, *B.fonsecai* can often be found among ferns (*Pteridiumarachnoides* (Kaulf.)) growing near forested areas (Frederico Menezes, pers. obs.), and occasionally in swamp areas. Only juveniles were found within a forest (about 150 m inside). This difference in habitat use may be related to milder temperatures and protection against visually oriented predators. The reproductive cycle has been described by Menezes et al. (in press). We observed the following defensive tactic behaviors: tail vibrating (against the substrate and its own body), cloacal discharge, hiding the head under the body coils and striking.

###### Altitudinal variation.

This species is found at a minimum of 400 m a.s.l. in Barra Mansa, RJ and a maximum of 1730 m a.s.l. in Campos do Jordão, SP (Bérnils 2009). The maximum altitudinal record for this study area is 2175 m a.s.l. in Itamonte, MG. Most of the toponyms where this species can be found (about 65%) are located at altitudes above 800 m a.s.l.

###### Distribution and habitat.

Southeastern Brazil (Minas Gerais, Rio de Janeiro, and São Paulo) (Peters and Orejas-Miranda 1970, Wallach et al. 2014). It occurs in mixed ombrophilous forests and adjacent natural fields (Campbell and Lamar 2004, Bérnils 2009).

##### Bothrops jararaca

Taxon classificationAnimaliaSquamataViperidae

(Wied, 1824)

D8F6AB4A-FBAD-56F2-8E58-517EB7DE11C1

Figure 4J

###### Natural history notes.

A species of medium size (*n* = 2), semi-arboreal and mainly nocturnal (Sazima 1992, Marques et al. 2001). In January, a recently road-killed adult male was found in the morning in a forested area. In March, an adult was seen at 10:40 h. above a rock outcrop at 2150 m a.s.l. near a forested area. When the observer approached, it fled into the forest. We did not obtain information on diet or reproduction from the observed specimen. Available information on diet from prior studies indicates that *B.jararaca* is a specialist, with ontogenetic variation. When juvenile, it often feeds on ectothermic prey (amphibians). This shifts to endothermic prey during adulthood (Sazima 1992). The reproductive cycle is seasonal and biennial. Pregnant females can be found from November to March (Almeida-Santos and Salomão 2002). Gestation ranges from 152 to 239 days, with fecundity from three to 36 snakelets (Alves et al. 2000, Almeida-Santos and Salomão 2002).

###### Altitudinal variation.

This species was found at a minimum of sea level between Rio Grande do Sul and Bahia with a maximum of 1640 m a.s.l. in Parque Nacional da Serra da Bocaina, SP (Bérnils 2009). The maximum altitudinal record for this species from this study area is an individual recorded at 2150 m a.s.l., in Baependi-MG. Of the surveyed toponyms, 33% occur at low elevations (0–400 m a.s.l.) and 41.5% at intermediate altitudes (400–800 m a.s.l.) (Bérnils 2009).

###### Distribution and habitat.

Central-west, northeast, southeast, and southern Brazil (Bahia, Espírito Santo, Mato Grosso, Minas Gerais, Paraná, Rio de Janeiro, Rio Grande do Sul, São Paulo, and Santa Catarina), Paraguay and Argentina (Wallach et al. 2014). This species is common in ombrophilous and seasonal forests, although it can also be found in secondary forests and disturbed areas (Bérnils 2009).

##### Bothrops neuwiedi

Taxon classificationAnimaliaSquamataViperidae

Wagler in Spix, 1824

DE647BF8-11D6-5281-836C-49B17871A096

Fig. 4 K

###### Natural history notes.

Medium-sized snake (*n* = 1), terrestrial and nocturnal (Marques et al. 2016). We spotted an adult during the day (12:50 h) in March, in a rocky field area. It was basking near a forest fragment at 2150 m a.s.l. When the observer approached, it fled into the forest. The available data in the literature indicates it was found mainly in fields and other open formations (Borges and Araújo 1998, Valdujo et al. 2002, Bérnils 2009). Diet is composed primarily of mammals (Martins et al. 2001, Valdujo et al. 2002).

###### Altitudinal variation.

The *neuwiedi* complex species was found at a minimum of sea level in the coast of Rio de Janeiro State, and a maximum of 1600 m a.s.l. in Parque Estadual do Ibitipoca, MG (Bérnils 2009). The maximum altitudinal record for this species is extended here, with an individual observed at 2150 m a.s.l. in the PESP, Baependi, MG. Bérnils (2009) states more than 80% of the surveyed toponyms are located in mountain and plateau areas, and only five toponyms have been recorded at sea level.

###### Distribution and habitat.

Northeast, central-west, southeast and southern Brazil (Bahia, Goiás, Minas Gerais, Paraiba, Paraná, Rio de Janeiro, São Paulo and Santa Catarina) (Wallach et al. 2014). Like other taxa of the *neuwiedi* complex, this species occurs in open formations, such as savannas, rocky fields, and steppes (Bérnils 2009).

##### Crotalus durissus

Taxon classificationAnimaliaSquamataViperidae

Linnaeus, 1758

C6852B44-983D-5676-A489-33FFA7797082

Figure 4L

###### Natural history notes.

Species of medium size (*n* = 1), with terrestrial and nocturnal habits (Marques et al. 2001). In January, an adult male was found during the day (13:00 h) in a high altitude grassland area. It was moving from the edge of a small forested area, towards an open field. Diurnal habits of this species have been described in reports by Sawaya et al. (2008) and Tozetti and Martins (2013) as well. No information of diet or reproduction was obtained from the specimen we observed. Available data indicates *Crotalus* has specialized in mammals, but may also ingest lizards (Sant’Anna 1999, Almeida-Santos and Germano 1996, Hoyos 2006). Interestingly, we found feces from an unidentified feline near the site of observation (at 2000 m a.s.l.) that contained a rattle and rattlesnake’s scales, indicating feline predation. Reproduction is viviparous, with a biennial reproductive cycle. Vitellogenesis starts in March and gestation goes through October and January and recruitment happens between January and March (Almeida-Santos and Salomão 1997, Almeida-Santos and Orsi 2002). This specimen presented the following defensive behaviors: cloacal discharge, rattle vibration, s-coil formation, and strike.

###### Altitudinal variation.

This species is found at a minimum of sea level for the coasts of Argentina, Uruguay, Rio Grande do Sul and Bahia and maximum of 1400 m a.s.l. at Taquaral Farm, Paraty, RJ (Bérnils 2009). The maximum altitudinal record for this species in this study area is an individual recorded at 1950 m a.s.l., in the Baependi, MG. Most of the surveyed toponyms occur at intermediate altitudes. Only 21% were found between 0–400 m a.s.l.; 51% between 401 and 800 m a.s.l. and 28% above 801 m a.s.l. (Bérnils 2009).

###### Distribution and habitat.

Southern and southeastern Brazil (Amapá, Amazonas, Bahia, Ceará, Goiás, Mato Grosso, Mato Grosso do Sul, Maranhão, Minas Gerais, Pará, Paraíba, Paraná, Pernambuco, Piauí, Rio de Janeiro, Rio Grande do Norte, Rio Grande do Sul, Rondônia, Roraima, Santa Catarina, São Paulo), Netherlands Antilles (Aruba), Guyana, Suriname, French Guiana, Peru, Colombia, Venezuela, Uruguay, Bolivia and Paraguay (Bérnils 2009, Wallach et al. 2014). This species is typically found in open formations, with little vegetation, such as savannas and steppes (Campbell and Lamar 2004).

## Discussion

### Species composition

The 24 snake species recorded in this study correspond to approximately 11% of the 219 species known from the phytogeographical domain of the Atlantic Forest (Moura et al. 2016). In general, the observed richness to the PESP is similar than that of other sites in the low-elevated Southeast Atlantic Forest (e.g., Marques and Sazima 2004, Centeno et al. 2008, Pontes and Rocha 2008, Hartmann et al. 2009a,b, São Pedro and Pires 2009, Moura et al. 2012, Trevine et al. 2014). Given the altitude increments of this sampling effort, several high-altitude species are expected to be found in the area, such as *Ditaxodontaeniatus* (Peters *in* Hensel, 1868), with a record to the Campos do Jordão - SP, approximately 100 km from the study area (see Thomas et al. 2006); *Philodryasarnaldoi* (Amaral, 1933), with two records for the Franca - SP, approximately 330 km away from the study site (Bérnils 2009). Despite the distance, Franca is directly connected to the extreme west of Serra da Mantiqueira (Bérnils 2009), so both places may present common elements in their faunas (R.S. Bérnils pers. comm.). Other possible species are (for details, see Bérnils 2009): *Micrurusdecoratus* (Jan, 1858) with a record for Caxambu – MG (ca. 27 km) (Gonzalez et al. 2014b); *Ptychophisflavovirgatus* Gomes, 1915, with a record for Liberdade - MG (ca. 45 km) (Gonzalez et al. 2014a); *Siphlophislongicaudatus* (Andersson, 1901), with a record for Munhoz - MG (ca 55 km); *Phalotrisreticulatus* (Perters, 1960), also for Munhoz - MG; *Erythrolamprusjaegeri* (Günther, 1858), recorded for Campos do Jordão – SP (ca. 100 km); *Pseudoboaserrana* Morato, Moura-Leite, Prudente & Bérnils, 1995, recorded for Bocaina de Minas – MG (ca. 35 km); *Echinantheraamoena* (Jan, 1863), recorded for Baependi – MG (ca. 25 km); *E.melanostigma* (Wagler 1824), recorded for Lambari - MG (ca. 65 km); *E.undulata* (Wied-Neuwied, 1824), recorded for Campos do Jordão - SP (ca. 100 km); *Taeniophallusbilineatus* (Fischer, 1885), recorded for Campos do Jordão - SP (ca. 100 km) and *T.persimilis* (Cope, 1869), recorded for Bananal - SP (ca. 70 km) (Bérnils 2009).

In any study, the relative frequency of snakes may be influenced by the sampling method (Marques 1998; Martins and Oliveira 1998). Here, the high frequency of *Atractuszebrinus* may be related to the use of pitfall traps, which accounted for 53% of records for that species. Nevertheless, disregarding pitfall trap records, *A.zebrinus* remains among the four most frequently observed species in the study area with seven records. Despite the considerable sample effort (2002 to 2007), Cardoso (2011) did not record *A.zebrinus* in a neighboring area (Farm Santa Elisa, Munhoz, MG, at 1320–1640 m a.s.l.). A possible explanation for this discrepancy is a lack of pitfall trappin method in that study effort. However, *A.zebrinus* has a confirmed voucher in the same farm in the Butantan Institute Collection (Frederico Menezes, pers. obs.). We propose this species simply may not be abundant in the study area covered by Cardoso (2011). In a similar discrepancy at Núcleo Curucutu, SP, Parque Estadual da Serra do Mar, Batista (2017) did not register *A.zebrinus* for this locality despite the use of pitfall traps. While, in an earlier study, Barbo et al. (2008) collected a specimen in the area. It is possible the altitudinal gradient influences species abundance, as noted by Lawton et al. (1987). *Atractuszebrinus* was the most abundant species in two inventories which were carried out at 1500 meters above the sea level: Ortiz et al. (2017), in Serra da Bocaina, with seven individuals (none captured by pitfall traps), and the present study, in Serra da Mantiqueira, with 15 individuals. However, this interpretation remains speculative and we suggest further tests, with a larger data set, to investigate this possibility.

In contrast to other Neotropical snake community studies (Cardoso 2011, Fiorillo 2016, Hartmann 2009a, b, Marques 1998, Cechin 1999, Sawaya et al. 2008, Costa et al. 2010), the family Viperidae was not the most abundant in this study. Our findins are more similar to the observations from temperate areas of Araucaria Forests and associated ecosystems (Di-Bernardo 2007, Deiques 2009). This may reflect a pattern for elevated areas. In this context, several factors may account for the predominance of *B.fonsecai* over other viperids (*B.jararaca*, *B.neuwiedi*, and *C.durissus*), which are somewhat common and easy to find (see Sazima 1988, Sazima 1992, Marques and Sazima 2004, Sawaya et al. 2008, Hartmann 2009a,b). Climatic factors, especially temperature, can act directly on the abundance of species that coexist in a given locality (Vitt 1987). We hypothesize that *B.fonsecai* is more tolerant of lower average temperatures, and as a result predominates in this study area where temperature may be a limiting factor for abundance of other vipers. For example, *B.jararaca* is very abundant in sites between 0 - 800 m altitude and yet rare in higher localities of Serra da Mantiqueira and Araucaria Plateau (Bérnils 2009). Altitude also seems to influence the population density of species *Chironiusbicarinatus*, *E.miliaris*, *O.clathratus*, *S.mikanii*, and *S.pullatus*. These species are well-represented in inventories carried out at lower altitudes (e.g., Hartmann et al. 2009a,b, Marques 1998, Fiorillo 2016, Trevine et al. 2014), but are rare in the study area, with only one record each. Regarding viperids *Bothropsfonsecai* and *B.alternatus* specifically, the later was not recorded in the PESP (1600 to 2359 m als). Hoge and Belluomini (1964) have discussed allopatry between these two species for the state of São Paulo and in areas near Minas Gerais State. Bérnils (2009) affirmed the allopatry proposed by these authors was not accurate, since, in these states some areas where *B.fonsecai* occur within areas where *B.alternatus* is dominant. However, sympatry could not be confirmed for these locations primarily because the specimens, housed in zoological collections, do not contain precise georeferences (Bérnils 2009). *Bothropsalternatus* is confirmed for Aiuruoca (IBSP data), at Ponte Coberta Farm (between 900 and 1000 m a.s.l.). In the same municipality, we recorded a *B.fonsecai* at approximately 1900 m a.s.l., which indicates sympatry between these species in the area. *B.fonsecai* is likely to be restricted to the higher altitudes, with different microclimatic conditions, and where there are still well-preserved forest fragments (Frederico Menezes pers. obs.). As for *B.alternatus*, this species is concentrated in lower open areas (Bérnils 2009).

The high species richness registered for the PESP might be related to environmental heterogeneity and the mix of habitats allowing more species of reptiles to coexist (Pianka 1969). However, comparing abundance data across altitude suggests this might have the greater effect on the number of individuals found. The frequency of individuals obtained in the PESP was at least 5.7 times lower than in Marques (1998), 3.5 times lower than Hartmann (2009a), 3.3 times lower than Fiorillo (2016), and only larger than Trevine et al. (2014). It is necessary to consider that these inventories were not performed uniformly and this could be a bias in comparing the abundance of snakes between these localities. Though decrease in abundance and species richness as altitude increases has been observed in other animal groups (Lawton et al. 1987, Moraes et al. 2017), it remains to be explored for snakes (Marques 1998). Undoubtedly, factors influencing species abundance across altitude are complex and differ for species and altitudinal transects. Although this hypothesis is speculative, we encourage further research to test this relationship in Brazilian snakes through systematic samplings at different altitudinal gradients.

### Comparison with other snake assemblages from the Atlantic Forest of southeastern Brazil

The species composition of the Parque Estadual da Serra do Papagaio is very similar to the Munhoz, which is also in southern Serra da Mantiqueira. These areas have very similar vegetation types. Both are present in the Alto Paraná Atlantic Forest, which contains rocky fields, savannahs, and montane forests (sensu Olson et al. 2001). The only other locality with similar vegetation to PESP and Munhoz is the São José do Barreiro, in São Paulo State (Ortiz et al. 2017). However, this locality belongs to another topographical complex: the Serra da Bocaina, which is separated from the Serra da Mantiqueira by the Paranapiacaba valley. This valley may pose a geographical barrier to the dispersal of the snake fauna (Ortiz et al. 2017, this study). Thus, the Serra da Mantiqueira seems to contain a *sui generis* composition of snake species, resulting in group 3 of our cluster analysis.

The other two main groups are composed of plateau areas (1) in the states of São Paulo and Minas Gerais (except localities in southern Serra da Mantiqueira); and (2) lowland areas and in São Paulo and Rio de Janeiro states. The formation of a cluster containing low coastal localities has already been described for anurans (Pombal Jr. et al. 2004, Bertoluci et al. 2007, Forlani et al. 2010). Coastal climatic conditions and other environmental characteristics may be the primary factors acting on formation of a particular fauna. These are certainly different between different areas of the Atlantic Forest (Dixo and Verdade 2006, Forlani et al. 2010). The presence of snake species found only in higher areas of the Atlantic Forest had already been highlighted by previous studies (e.g., Bérnils 2009). Thus, a more significant similarity was expected between these highlands. This is especially true of the Planalto Paulista, due to the number of localities inserted in the Ecological Continuous of Serra de Paranapiacaba, one of the largest preserved fragments of Atlantic Forest in São Paulo State (Pisciotta 2002).

### Conservation

The Serra da Mantiqueria is defined as an area of particular biological importance, and it stands out in the region as one of the most important areas for biodiversity conservation in the state of Minas Gerais. This status is justified by the high abundance of rare and threatened species (Drummond et al. 2005). The Parque Estadual da Serra do Papagaio is located within the Serra da Mantiqueiria and carries many different types of vegetation, including fields, forests, and ombrophilous mixed forest (Olson et al. 2001, Silva et al. 2001). As it is one of the few areas in Minas Gerais protecting this variety of vegetation, it is regarded as an area of extreme biological importance (Silva et al. 2008). Currently, the mixed ombrophilous forest has been reduced to less than 5% of its original size (SOS Mata Atlântica and INPE, 2014). These remaining areas constitute the last refuges, sheltering several high altitude species (Backes 2009, Cardoso 2011). *Bothropsfonsecai* is endemic to an area that covers approximately 120,000 km^2^, in the mountainous portions of São Paulo, Minas Gerais, and Rio de Janeiro States (Barbo 2012). Of the viper species found in this study, *B.fonsecai* was the only one which was not located outside the PESP limits. It appears to be relatively common in protected areas, but rare in unprotected areas. The absence of this species in disturbed areas suggests it may be sensitive to environmental degradation and/or may benefit from the protection. Either of these contributes to a critical situation that demands immediate efforts for conservation. Additionally, little is known about the ecology of *B.fonsecai*. Although we have collected field data about its natural history, a detailed study is still necessary to describe its displacements, home range, habitat use, and reproduction (e. g. Hartmann et al. 2003, Tozetti and Martins 2008).

The dipsadid *Mussuranamontana* is another species of interest found in this area. This species is endemic to a region that covers about 16,000 km^2^ in the mountainous portions of São Paulo, Minas Gerais, and Rio de Janeiro states (Barbo 2012). According to Costa et al. (2015), it is susceptible to habitat disturbance. In the national assessment of the Brazilian fauna risk of extinction, *M.Montana* was classified as “least concern” (MMA 2014). Regional classification of *M.montana* is “vulnerable” and “near threatened” in the states of São Paulo and Minas Gerais, respectively (Biodiversitas 2007, Bérnils et al. 2009). Recently, Costa et al. (2015) presented three new records of this species in protected areas of the State of Minas Gerais, increasing its known range considerably and supporting the lower conservation status in Minas Gerais. *M.montana* is known only to inhabit tropical ombrophilous or seasonal forest formations of montain ranges from Bocaina, Mantiqueira and Órgãos, between 750–1,610 m a.s.l. (Bérnils 2009, Costa et al. 2015). However, sampling in potentially suitable areas for the species did not yield encounters (Costa et al. 2015). This may mean that *M.montana* is present over a smaller range and more specific habitat than was previously expected. If true, this reinforces the need for conservation in areas where it occurs.

The present study provides data on the occurrence and natural history of snakes in the Parque Estadual da Serra do Papagaio. However, additional field research is encouraged. New inventories could contribute additional knowledge about the natural history of the species of this region, especially those of which ecological, biological, and morphological data are scarce, such as *Mussuranamontana*. Our ordination analysis shows a remarkable difference between the compositions of snake faunas between different altitudinal gradients in the Brazilian Atlantic Rainforest. The Serra da Mantiqueira, where the Parque Estadual da Serra do Papagaio is located, seems to present a very unique composition of snake species, most of which are not shared with others localities from the southeast of Brazil. The diverse vegetation types found in this region permits the existence and maintenance of diverse species of animals. Additionally, the protected areas belonging to the Atlantic Forest affords greater potential to find rare and endemic species. These facts together make an argument for prioritizing the conservation of this park. Nevertheless, even if recognized as a conservation unit, the park continues to suffer depredation of its flora and fauna, mainly through illegal hunting. Regrettably, this alarming scenario is widely observable in Brazil due to the scientific scrapping and the neglect of the Brazilian government to control its natural resources (Bockmann et al. 2018).

### Key to the snake species from Serra do Papagaio State Park – MG

**Table:** 

1	Loreal pit present; solenoglyphous dentition	** Viperidae **
–	Loreal pit absent, aglyphous or opisthoglyphous	**2**
2	Aglyphous dentition; even number of dorsal scale rows	** Colubridae **
–	Aglyphous or opisthoglyphous dentition; odd number of dorsal scale rows	** Dipsadidae **


### Viperidae

**Table:** 

1	Rattle at the tip of the tail; with some enlarged shields on top of the head	*** Crotalus durissus ***
–	No rattle at the tip of the tail; tiny shields on top of the head	**2**
2	Inverted v-shaped spots along the dorsum; second supralabial fusioned with the prelaculal	*** Bothrops jararaca ***
–	Trapezoid spots along the dorsum; second supralabial not fused with the prelaculal	**3**
3	Non-fragmented trapezoid spots along the dorsum; black venter	*** Bothrops fonsecai ***
–	Trapezoid spots are fragmented at the midline to ventrals; venter cream, with several tiny brown spots	*** Bothrops neuwiedi ***

### Colubridae

**Table:** 

1	12 scale rows at mid-body	**2**
–	More than 12 scale rows at mid-body	*** Spilotes pullatus ***
2	2-4 rows of keeled dorsal scales; first third of body black or dark gray, vertebral stripe yellowish or cream; top of the head tan to brown (distinct from the background color of the body); venter cream, with black bordered scales	*** Chironius brazili ***
–	Two rows of keeled dorsal; olive green at the first third of the body; head color similar to the body; venter yellow, without black bordered scales	*** Chironius bicarinatus ***

### Dipsadidae

**Table:** 

1	17 or fewer scale rows at mid-body	**2**
–	19 scale rows at mid-body	**10**
2	15 scale rows at mid-body	**3**
–	17 scale rows at mid-body	**5**
3	Internasal shields absent; body almost uniformly red, with black tail	*** Apostolepis assimilis ***
–	Internasal shields present; without red color on the dorsum	**4**
4	Black eyes, indistinguishable pupil; venter white, with black spots	*** Sibynomorphus mikanii ***
–	Eyes with brown background color and round pupil, easily distinguishable; venter immaculate yellow	** Taeniophallus gr. occipitalis **
5	Dark lining, loreal shield absent	*** Tomodon dorsatus ***
–	Light lining, loreal shield present	**6**
6	Dorsal color brown, with a lighter longitudinal line on each side; three supralabials in contact with the eyeball	*** Gomesophis brasiliensis ***
–	Coloration not as above; two supralabials in contact with the eyeball	**7**
7	Dorsal scales with black borders and light centers; venter cream yellow, base of ventral scales with black edges	*** Erythrolamprus miliaris ***
–	Coloration not as above	**8**
8	Stout and short body; small eyes	*** Atractus zebrinus ***
–	Slender and elongate body; large eyes	**9**
9	Fourth row of dorsal scales with white dots that, together, form a continuous line along the body; brown top of the head and body; ventral scales with transverse band	*** Echinanthera cephalostriata ***
–	The fourth row of dorsals without white dots; top of the head black, contrasting with the brown body; ventral scales without transverse band	*** Taeniophallus affinis ***
10	Two apical pits	**11**
–	Single apical pit	**14**
11	Red iris	**12**
–	Black or dark brown irish, never red	**13**
12	Juveniles are brick red, with a dark brown longitudinal vertebral stripe; adults are entirely dark brown	*** Mussurana montana ***
–	Coral color pattern, with black triangular spots, bordered with white, background color red	*** Oxyrhopus rhombifer ***
13	Loreal shield usually absent; contact between frontal and preocular absent	*** Oxyrhopus clathratus ***
–	Loreal shield present; contact between frontal and preocular present	*** Boiruna maculata ***
14	Keeled dorsal scales; dorsum uniform green	*** Philodryas aestiva ***
–	Smooth dorsal scales; dorsum never green	**15**
15	*Canthusrostralis* very evident; without postocular stripe	*** Philodryas patagoniensis ***
–	*Canthusrostralis* not evident; conspicuous postocular stripe	**16**
16	With two large post-diastemal fangs, aglyphous; venter cream, without longitudinal lines	*** Xenodon merremii ***
–	Without two large post-diastemal fangs, but opisthoglyphous; venter cream, with two to four longitudinal lines	*** Thamnodynastes strigatus ***

## Supplementary Material

XML Treatment for Chironius bicarinatus

XML Treatment for Chironius brazili

XML Treatment for Spilotes pullatus

XML Treatment for Apostolepis assimilis

XML Treatment for Atractus zebrinus

XML Treatment for Boiruna maculata

XML Treatment for Echinanthera cephalostriata

XML Treatment for Erythrolamprus miliaris

XML Treatment for Gomesophis brasiliensis

XML Treatment for Mussurana montana

XML Treatment for Oxyrhopus clathratus

XML Treatment for Oxyrhopus rhombifer

XML Treatment for Philodryas aestiva

XML Treatment for Philodryas patagoniensis

XML Treatment for Sibynomorphus mikanii

XML Treatment for Taeniophallus affinis

XML Treatment for Taeniophallus gr. occipitalis

XML Treatment for Thamnodynastes strigatus

XML Treatment for Tomodon dorsatus

XML Treatment for Xenodon merremii

XML Treatment for Bothrops fonsecai

XML Treatment for Bothrops jararaca

XML Treatment for Bothrops neuwiedi

XML Treatment for Crotalus durissus
